# Supplementary material for: Algorithmic Assessments in Deciding on Voluntary, Assisted or Involuntary Psychiatric Treatment
Source: Diagnostics (Basel). 2022 Jul 26;12(8):1806. doi: 10.3390/diagnostics12081806 (PMC9330761; doi:10.3390/diagnostics12081806)
Supplement: Supplementary file 1 [file diagnostics-12-01806-s001.zip › diagnostics-1787209-supplementary/Supplementary Materials 2.pdf]

Ms. PaNu

Ms. PaNu is a 38-year-old female who presents to a general medical practice for an opinion regarding her future treatment on account of an allergic drug reaction to the use of carbamazepine for a diagnosis of Bipolar I Disorder. Ms. PaNu consulted a dermatologist the previous year who treated her condition with corticosteroid medication. Earlier this year she has been placed on lamotrigine that promptly elicited an exfoliative skin reaction again. Except her dermatological concerns Ms. PaNu, she has no other health problems in addition to the Bipolar I disorder.

Ms. PaNu has no history of any substance use disorder. She also has no history of any other medical conditions. Her physical health parameters are within normal limits on physical examination.

On clinical examination Ms. PaNu presents with an expansive mood, racing thoughts and pressured speech. She believes that she is “the best worker they have” at her place of employment despite two letters of warning in the past month. The physician notices that her self-esteem seems inflated. She tells the physician that she’s had “the best sex of my life” on multiple occasions over the past two weeks with different men she met at a club at different times. She is not in a romantic relationship with any of them. She does not consider this behaviour unsafe because she “trusts them all.” Her ability to concentrate during the interview is poor as is her short-term memory on account of her distractible thoughts. Her thoughts are racing so much that she cannot order her thoughts coherently. For the past week she experienced a decreased need for sleep according to her report.

Ms. PaNu tells the physician in confidence that she is “blessed”. She is hearing a mixture of “voices”, some of them unknown to her and others those of her deceased relatives, for the past week. She believes that she is “a special vehicle” through whom the “dead communicates with the world”. The “voices” seem so real that she is unshakeably convinced of a (supernatural) “force” that entered her mind and now controls her thoughts. All these symptoms occurred together, and she noticed these for the first time about two weeks ago.

The physician assesses Ms. PaNu as suffering from a mood disorder of severe degree. By this opinion she requires hospital admission and treatment to protect her wellbeing and reputation against the consequences of her behaviour. She does not belief that she is mentally ill at all and refuses to accept further psychiatric treatment as she “does not need it”. She is perplexed that the physician even suggests it. She is convinced that she is “blessed”. She decides not to stay for the hospital admission and prepares to leave the office of the physician. Her family is of the opinion that she requires admission.

Apply the algorithm in answering the question at this time: what would be the most suitable decision that is justified by the information actually provided: a) hospital admission in terms of the Mental Health Care Act is legitimately declined or not applicable; or b) hospital admission in terms of the Mental Health Care Act should proceed by which suitable legal status?
