# Supplementary figures and images for: Algorithmic Assessments in Deciding on Voluntary, Assisted or Involuntary Psychiatric Treatment
Source: Diagnostics (Basel). 2022 Jul 26;12(8):1806. doi: 10.3390/diagnostics12081806 (PMC9330761; doi:10.3390/diagnostics12081806)

Algorithm: *Incapacity and Legal State Assessment Algorithm (ILSAA)*

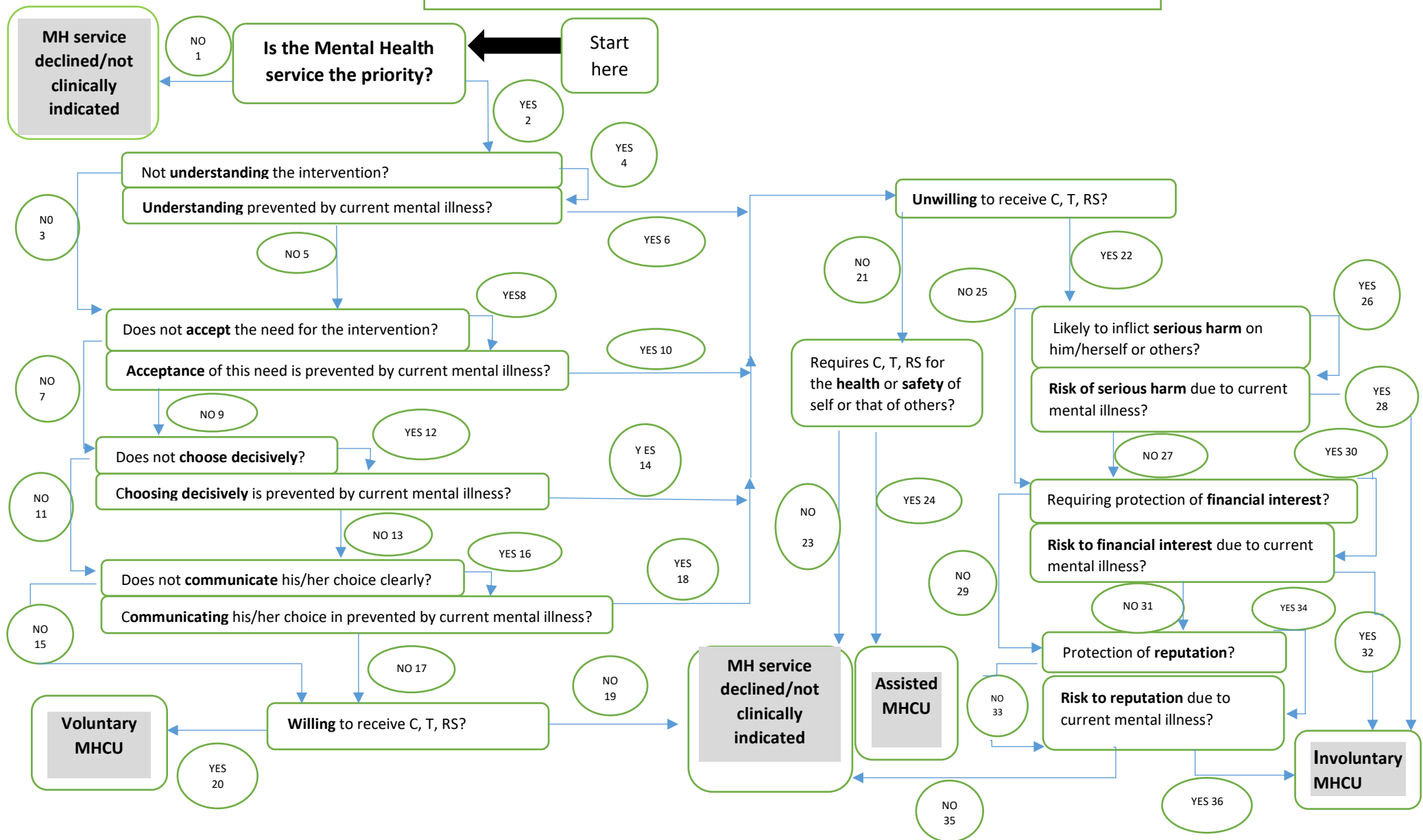

Supplement: Supplementary file 1 [file diagnostics-12-01806-s001.zip › diagnostics-1787209-supplementary/Supplementary Materials 1.pdf]
